# A Novel Finite Element-Based Method for Predicting the Permeability of Heterogeneous and Anisotropic Porous Microstructures

**DOI:** 10.3390/ma17122873

**Published:** 2024-06-12

**Authors:** Paris Mulye, Elena Syerko, Christophe Binetruy, Adrien Leygue

**Affiliations:** École Centrale Nantes, Nantes Université, CNRS, GeM, UMR 6183, F-44000 Nantes, France; paris.mulye@gmail.com (P.M.); elena.syerko@ec-nantes.fr (E.S.); adrien.leygue@ec-nantes.fr (A.L.)

**Keywords:** finite element method, homogenization, microstructure, porous media, permeability tensor, anisotropy, heterogeneity, boundary condition

## Abstract

Permeability is a fundamental property of porous media. It quantifies the ease with which a fluid can flow under the effect of a pressure gradient in a network of connected pores. Porous materials can be natural, such as soil and rocks, or synthetic, such as a densified network of fibres or open-cell foams. The measurement of permeability is difficult and time-consuming in heterogeneous and anisotropic porous media; thus, a numerical approach based on the calculation of the tensor components on a 3D image of the material can be very advantageous. For this type of microstructure, it is important to perform calculations on large samples using boundary conditions that do not suppress the transverse flows that occur when flow is forced out of the principal directions. Since these are not necessarily known in complex media, the permeability determination method must not introduce bias by generating non-physical flows. A new finite element-based method proposed in this study allows us to solve very high-dimensional flow problems while limiting the biases associated with boundary conditions and the small size of the numerical samples addressed. This method includes a new boundary condition, full permeability tensor identification based on the multiscale homogenization approach, and an optimized solver to handle flow problems with a large number of degrees of freedom. The method is first validated against academic test cases and against the results of a recent permeability benchmark exercise. The results underline the suitability of the proposed approach for heterogeneous and anisotropic microstructures.

## 1. Introduction

Permeability is an average medium property that measures the ability of the porous medium to transport the fluid. Permeability is defined as a scalar value for isotropic porous media but can be defined as a tensor to reflect its dependence on the orientation of the porous structure relative to the coordinate system. Two types of permeability are defined according to the type of flow in the porous medium [1]. The saturated permeability is the permeability of a porous medium saturated by one fluid. It only depends on the geometrical pore space of the material. The effective permeability with respect to the liquid phase is the permeability of a porous medium as experienced by that liquid. It includes the interfacial and wetting properties. This article will focus on saturated permeability.

In science and engineering, permeability is a very important physical property of porous media because it controls the directional movement and flow rate of fluids in the porous structure. Porous materials can be natural, such as soil and rock, or synthetic, such as a dense network of fibres or open-cell foams [2]. Permeability is influenced by factors such as the pore structure, through its size distribution and tortuosity, and the type of permeating fluid. Tortuosity describes the sinuosity of the pore space relating the actual flow path length to the straight distance between the ends of this path and is the intrinsic geometric property of the pore space.

The applications of permeability are diverse and include various fields such as civil engineering, pavement engineering, environmental engineering, petroleum engineering, geotechnical engineering, tissue engineering, agricultural engineering, and materials engineering, to name a few. For example, permeability is required for the design and construction of various civil engineering projects such as underground structures, dams, and reservoirs. It helps engineers to predict how soil behaves when subjected to various loads and water pressures. Permeability is simple in concept but has some very complex aspects in practice, especially when trying to make accurate measurements or predictions.

The manufacturing of composite materials, whether for semi-finished products such as prepregs or finished parts, relies on impregnation by the resin of a dense network of fibres. The latter can be viewed as a porous medium. Controlling the transfer of a liquid resin within this medium is essential to obtain defect-free materials and parts. The knowledge of the permeability of the fibre network is generally required to adjust manufacturing parameters such as pressure, flow rate, and temperature.

The slender form factor of the fibres and the preferred orientations they assume in many engineered parts result in a fairly pronounced directionality of the flows. In addition, optimizing part design for the highest mechanical performance-to-mass ratio means varying the orientations and even the volume fraction of the fibres in the part. The fibre network becomes heterogeneous and anisotropic throughout the part. Measuring the components of the permeability tensor becomes difficult when characterizing fibrous preforms where the arrangement of the fibres changes spatially, such as materials produced by automated fiber placement. Due to the high cost and long duration of such experiments, it is attractive to determine the permeability by numerical simulation.

Compared to granular porous media whose permeability is often isotropic, the permeability of fibrous networks is almost never isotropic. Anisotropy is also observed in fractured porous rocks, where a dual-scale flow occurs in the network of interconnected fractures and in the porous rock itself. The permeability of such a medium results from the interaction of flows within the two interconnected networks. In this paper, we focus on porous media with heterogeneous but monodisperse porosity, which can lead to anisotropic permeabilities. The single phase flow in such material is investigated here.

The general approach of computing the permeability of a porous material is to first solve the stationary Stokes equations to obtain the pressure and/or velocity fields in the computational domain and then to average the computed fields by an appropriate permeability identification technique. Different numerical methodologies for the solution of this problem with different discretisation techniques, physical variables formulation, boundary conditions, etc., exist in the literature. A recent benchmark on the image-based permeability determination of fibrous materials has illustrated this diversity of numerical approaches [3]. The most widely used numerical approximation methods are the Finite Volume Method (FVM) [4] and the Finite Element Method (FEM) [5]. Other methods, such as the Finite Difference Method (FDM) [6], Pore Networks Modelling (PNM) [7], the Lattice Boltzmann Method (LBM) [8,9], or the Smoothed Particle Hydrodynamics (SPH) method [10], can also be used for flow field calculation. In [11], the three numerical methods LBM, SPH, and FEM were compared for pore-scale resolved simulations on a simple 2D benchmark problem. The results are also compared with experimental measurements and analytical determination. The three methods give very similar results, but it is pointed out that they are limited by computational cost. This conclusion is consistent with the results of the benchmark study [3], where it is shown that the choice of numerical discretisation method itself is not a dominant factor influencing the permeability prediction accuracy.

Another important aspect to consider when modelling complex geometries in multi-dimensional problems is that in two and three dimensions, curvature is handled more naturally in FVM and FEM than in FDM due to the integral nature of the equations used. In the case of the FVM in a topologically regular mesh, the flux calculation is quite straightforward, which is different for an irregular mesh (e.g., in an automatically generated tetrahedral mesh). This shortcoming is absent in the finite element method: the same effort for the calculation by FEM is needed for the regular geometries and for the complex ones. Another advantage of FEM, as opposed to the FVM, is that there is no need to formulate additional boundary conditions with the presence of natural boundary conditions in the discretized equations. In SPH, since there is no mesh and no need to calculate fluxes, the method works well with complex moving geometries. However, the implementation of inlets, outlets and pressure boundary conditions is not as straightforward as in FEM and FVM. In another meshless method, LBM, since fibrous reinforcements often represent the degenerated domains (with one dimension smaller than the others), the necessity to use a regular LBM lattice leads to an extremely CPU time-consuming calculation, even with a modest spatial resolution of the lattice, as was shown in [9]. The PNM often uses idealized pore shapes compared to the natural porous media, which affects the accuracy of the model, especially for complex systems such as fibrous media. When pores have more complex shapes that deviate from spheres, ellipsoids, and tubes, another more accurate approach is to skeletonise the media to retain much of the topological information of the real media [12].

The application of appropriate boundary conditions and the used permeability identification technique are much more influencing in the accuracy of the permeability determination. The boundary conditions applied in the computational domain must not suppress transverse flow, which can occur naturally if the flow is not forced along the principal directions of the porous medium [13]. Due to the heterogeneity of numerous materials, permeability calculations should be performed on a representative volume, which should be large enough to account for all geometric characteristics of the medium. Challenges then arise regarding the efficiency of numerical problem solving and the amount of memory required.

## 2. Objectives and Content of the Study

The literature review shows that the main challenge in the numerical determination of permeability lies in the ability to process numerical samples of sufficient size to limit the biases associated with strong assumptions about the representativeness of the medium under consideration and inappropriate boundary conditions that create unphysical flows.

In view of the above, since the porous materials considered are generally anisotropic and heterogeneous, the main objectives of this work are as follows:

- To propose and investigate a finite element-based numerical methodology that can predict the homogenized permeability of heterogeneous and anisotropic porous microstructures where the flow in the microstructure is induced by a body force.
- To demonstrate the necessity to calculate the full permeability tensor including its off-diagonal components in order to capture the influence of the local anisotropy.

The article is organized as follows. In Section 3, the pore-scale resolved flow model and the permeability determination method are described. In Section 4, the novel finite element-based method for predicting the permeability of heterogeneous and anisotropic porous microstructures is validated against three academic benchmark cases. More complex and representative cases are addressed in Section 5.1, where the results are discussed against those of a benchmark problem. The specific question of the effect of the directionality of porous media on the determination of the components of the permeability tensor is discussed in Section 5.2.

## 3. Modelling of Single-Phase Steady-State Porous Media Flow at Micro Scale

In this work, a fully saturated porous medium occupying the space $\Omega \in R^{n}$ is considered to be made up of up to two non-overlapping phases, one representing a rigid, non-permeable solid ($\Omega_{s}$) and the other representing an incompressible linear viscous fluid ($\Omega_{f}$). The steady-state problem of flow [14] through such porous media decribed by the Stokes equation can be formulated as follows:

(1) $$\begin{array}{cccc} \nabla \;\cdot \;\sigma & =b & \mathrm{in}\;\Omega_{f}\; & (\mathrm{Balance}\;\mathrm{of}\;\mathrm{linear}\;\mathrm{momentum}) \end{array}$$

(2) $$\begin{array}{cccc} \nabla \;\cdot \;v & =0 & \mathrm{in}\;\Omega_{f}\; & (\mathrm{Incompressibility}\;\mathrm{constraint}) \end{array}$$

(3) $$\begin{array}{cccc} \sigma & =-pI+2\mu \nabla^{s}v & \mathrm{in}\;\Omega_{f}\; & (\mathrm{Constitutive}\;\mathrm{model}\;\mathrm{for}\;\mathrm{linear}\;\mathrm{viscous}\;\mathrm{fluid}) \end{array}$$

(4) $$\begin{array}{cccc} v & =0 & \mathrm{in}\;\Omega_{s}\; & (\mathrm{Impermeable}\;\mathrm{solid}) \end{array}$$

(5) $$\begin{array}{cccc} v & =v_{D} & \mathrm{in}\;\Gamma_{f}^{D}\; & (\mathrm{Dirichlet}\;\mathrm{boundary}\;\mathrm{conditions}\;\mathrm{for}\;\mathrm{fluid}) \end{array}$$

(6) $$\begin{array}{cccc} n\;\cdot \;\sigma & =t & \mathrm{in}\;\Gamma_{f}^{N}\; & (\mathrm{Neumann}\;\mathrm{boundary}\;\mathrm{conditions}\;\mathrm{for}\;\mathrm{fluid}) \end{array}$$

(7) $$\begin{array}{cccc} v_{D} & =0 & \mathrm{in}\;\Gamma_{s}\; & (\mathrm{No}-\mathrm{slip}\;\mathrm{boundary}\;\mathrm{conditions}\;\mathrm{on}\;\mathrm{solid}\;\mathrm{interfaces}) \end{array}$$

where $\Gamma_{f}^{D}$ indicates the fluid domain boundary on which Dirichlet conditions are imposed, $\Gamma_{f}^{N}$ is the fluid domain boundary where Neumann conditions are imposed, $\Gamma_{s}$ is the boundary of the solid domain, σ is the Cauchy stress tensor, b is the body force, I is the second order identity tensor, μ is the dynamic viscosity of the fluid, *p* is the mechanical definition of the pressure, t is the imposed boundary traction, n is the unit normal vector on the Neumann boundary, $v_{D}$ is the imposed velocity on Dirichlet boundary of the fluid domain, and $\nabla^{s}v$ is the strain rate defined by the symmetric part of the gradient of the velocity as

(8) $$\begin{array}{c} \nabla^{s}v=\frac{1}{2}\left[\nabla v+{\left(\nabla v\right)}^{T}\right] \end{array}$$

In the literature, several studies have demonstrated the use of a penalty-based approach [15,16]. It involves a relaxation of the incompressibility condition with a pseudo-compressibility condition, as follows:

(9) $$\begin{array}{cccc} \nabla \;\cdot \;v & =-\frac{p}{\lambda } & \mathrm{in}\;\Omega_{f}\; & (\mathrm{Pseudo}-\mathrm{compressibility}\;\mathrm{constraint}) \end{array}$$

where λ is a penalty parameter. The convergence of the numerical solution using a penalty-based approach has been demonstrated in [17]. The benefit of using the penalty-based approach is that it uncouples pressure and velocity. With this, one can solve the momentum equations in terms of only the velocity field without considering additional degrees of freedom (DoFs) associated with the pressure.

Despite the limitations and challenges associated with the choice of value for the penalty parameter itself, as discussed in [15], the reduction in the total number of degrees of freedom to solve is attractive for computationally costly problems such as the one in the virtual permeability benchmark [3] discussed further in Section 5.1. Thus, with the computational cost in mind, a modelling choice was made to use the penalty-based approach in this work.

### 3.1. PoroS 1.0: An Image-Based Stokes Flow Solver

**Figure 1:**
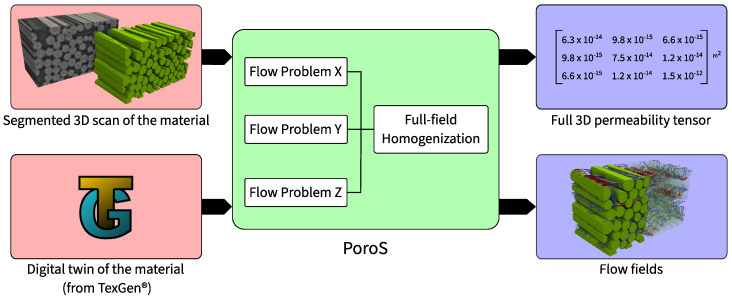
Overview and scope of PoroS: The input can be either a segmented 3D scan of the material or a digital twin of the material generated using TexGen^®^. The scope of PoroS consists of the Stokes flow problem solver and a full-field homogenization subroutine. Thus, the output of the solver consists of flow fields of the flow problems and a full 3D permeability tensor.

A software suite named `PoroS’ consisting of new voxel- (3D) or pixel-based (2D) porous media permeability solvers has been developed that uses a finite element approach (Figure 1). The finite element method is the most widely used numerical solution to engineering problems for the design and analysis of composite structures [18,19].

The numerical architecture of the solver has been specifically designed so that the following conditions are met:

- It can directly read the segmented images of the microstructure obtained from the micro-CT scan and perform the flow and permeability computations on them.
- It can also directly read a voxelized geometry of a digital twin of a material generated using the widely used open-source software TexGen^®^ v3.13.1 [20,21].
- It can handle flow problems with a large number of degrees of freedom (order of billions of DoFs).

Although PoroS contains several memory-efficient solvers to solve the linear system of equations, the results presented in this work use, in particular, one of the solvers which is a global matrix free preconditioned conjugate gradient solver.

### 3.2. Weak Form

Following the finite element approach with penalty, the weak form for the problem described in Section 3 reads as follows:

(10) $$\begin{array}{c} \underset{\mathrm{Viscous}\;\mathrm{Term}}{\underbrace{\int_{\Omega }\mu \;\left(\nabla w:\nabla v\right)\;d\Omega }}+\underset{\mathrm{Penalty}\;\mathrm{Term}}{\underbrace{\int_{\Omega }\lambda \;\left(\nabla \;\cdot \;w\right)\;\left(\nabla \;\cdot \;v\right)\;d\Omega }}=\underset{\mathrm{Body}\;\mathrm{Force}\;\mathrm{Term}}{\underbrace{\int_{\Omega }w\;\cdot \;b\;d\Omega }}+\underset{\mathrm{Neumann}\;\mathrm{Boundary}\;\mathrm{Term}}{\underbrace{\int_{\Gamma_{f}^{N}}w\;\cdot \;t\;d\Gamma }} \end{array}$$

where w is the test function for velocity such that

(11) $$\begin{array}{c} w\in V:=H_{d\Omega_{f}^{D}}^{1}\left(\Omega \right)=\left\{w\in H^{1}\left(\Omega \right)\;|\;w=0\;\mathrm{on}\;d\Omega_{f}^{D}\right\} \end{array}$$

### 3.3. Discretisation

In PoroS, a voxel-based approach (pixel-based in 2D) is followed. For 3D, a 27-node Taylor–Hood hexahedral element (HEX27) is implemented for the resolution of the velocity field. Similarly, for 2D, a 9-node quadrilateral element (QUAD9) is implemented [14,15,22]. From here onwards, the derivations are presented in 3D formulation; however, equivalent development is performed for 2D formulation as well.

The velocity vector at an arbitrary point $q\in \Omega_{f}$ is then given by

(12) $$\begin{array}{c} \left[\begin{array}{c} \underset{\left(3\times 1\right)}{V_{q}} \end{array}\right]=\sum_{i=1}^{27}\left[\begin{array}{ccc} N_{i} & 0 & 0\\ 0 & N_{i} & 0\\ 0 & 0 & N_{i} \end{array}\right]\left[\begin{array}{c} V_{i}^{x}\\ V_{i}^{y}\\ V_{i}^{z} \end{array}\right]=\left[\begin{array}{c} \underset{\left(3\times 81\right)}{N} \end{array}\right]\left[\begin{array}{c} \underset{\left(81\times 1\right)}{V_{e}} \end{array}\right] \end{array}$$

where $N_{i}$ are the classical shape functions corresponding to the HEX27 element evaluated at point q, and $V_{e}$ is the nodal velocity vector of the element.

### 3.4. Linear System of Equations

Using (12), the Equation (10) can be transformed into a linear system of equations as follows:

(13) $$\begin{array}{c} \left[A^{v}+A^{\lambda }\right]\left[V\right]=\left[F\right] \end{array}$$

where $A^{v}$ is the global viscous stiffness matrix and $A^{\lambda }$ is the global penalty stiffness matrix assembled from the elemental stiffness matrices $A_{e}^{v}$ and $A_{e}^{\lambda }$, respectively. They are defined as

(14) $$\begin{array}{cc} A_{e}^{v} & =\int_{\Omega_{e}^{f}}{B_{v}}^{T}\;D\;B_{v}\;d\Omega_{e}^{f} \end{array}$$

(15) $$\begin{array}{cc} A_{e}^{\lambda } & =\int_{\Omega_{e}^{f}}{B_{\lambda }}^{T}\;\lambda \;B_{\lambda }\;d\Omega_{e}^{f} \end{array}$$

In the latter, the strain rate (B) matrices are defined as

(16) $$\begin{array}{cccc} \underset{\left(6,81\right)}{B_{v}} & =\left[\begin{array}{cccc} \underset{\left(6,3\right)}{B_{v}^{1}} & \underset{\left(6,3\right)}{B_{v}^{2}} & \cdots & \underset{\left(6,3\right)}{B_{v}^{27}} \end{array}\right] & \underset{\left(6,81\right)}{B_{\lambda }} & =\left[\begin{array}{cccc} \underset{\left(6,3\right)}{B_{\lambda }^{1}} & \underset{\left(6,3\right)}{B_{\lambda }^{2}} & \cdots & \underset{\left(6,3\right)}{B_{\lambda }^{27}} \end{array}\right] \end{array}$$

(17) $$\begin{array}{cccc} \underset{\left(6,3\right)}{B_{v}^{i}} & =\left[\begin{array}{ccc} \frac{\partial N_{i}}{\partial x} & 0 & 0\\ 0 & \frac{\partial N_{i}}{\partial y} & 0\\ 0 & 0 & \frac{\partial N_{i}}{\partial z}\\ \frac{\partial N_{i}}{\partial y} & \frac{\partial N_{i}}{\partial x} & 0\\ 0 & \frac{\partial N_{i}}{\partial z} & \frac{\partial N_{i}}{\partial y}\\ \frac{\partial N_{i}}{\partial z} & 0 & \frac{\partial N_{i}}{\partial x} \end{array}\right] & \underset{\left(6,3\right)}{B_{\lambda }^{i}} & =\left[\begin{array}{ccc} \frac{\partial N_{i}}{\partial x} & \frac{\partial N_{i}}{\partial y} & \frac{\partial N_{i}}{\partial z}\\ \frac{\partial N_{i}}{\partial x} & \frac{\partial N_{i}}{\partial y} & \frac{\partial N_{i}}{\partial z}\\ \frac{\partial N_{i}}{\partial x} & \frac{\partial N_{i}}{\partial y} & \frac{\partial N_{i}}{\partial z}\\ 0 & 0 & 0\\ 0 & 0 & 0\\ 0 & 0 & 0 \end{array}\right] \end{array}$$

and the viscous 3D constitutive matrix D is a diagonal matrix given by

(18) $$\begin{array}{cc} \underset{\left(6,6\right)}{D} & =\mu \;\mathrm{diag}(2,2,2,1,1,1) \end{array}$$

In (13), the term $\left[V\right]$ corresponds to the global velocity nodal vector, whereas the term $\left[F\right]$ corresponds to the external force, which in the scope of this work is applied in the form of a body force similar to gravity. It is given by

(19) $$\begin{array}{c} \underset{\left(81,1\right)}{F}=\int_{\Omega_{e}^{f}}{\left[\begin{array}{c} \underset{\left(3\times 81\right)}{N} \end{array}\right]}^{T}\left[\begin{array}{c} \underset{\left(3\times 1\right)}{b} \end{array}\right]d\Omega_{e}^{f} \end{array}$$

where $\left[\begin{array}{c} b \end{array}\right]={\left[\begin{array}{ccc} b_{x} & b_{y} & b_{z} \end{array}\right]}^{T}$ is a vector denoting the body force.

### 3.5. Permeability Determination Procedure

The permeability tensor of a saturated porous medium (K) is associated with Darcy’s law, which relates the pressure gradient (∇P) to the volume-averaged fluid velocity (v) as follows:

(20) $$\begin{array}{c} v=-\frac{K}{\mu }\nabla P \end{array}$$

K is a second-order symmetric, positive-definite tensor, which is an intrinsic property of the porous medium. The emergence of the Darcy equation from the Stokes equation is well-justified by multi-scale asymptotic homogenisation [23] or averaging techniques [24]. In full-field numerical methods, the permeability tensor is obtained by solving a series of incompressible single-phase flow problems in a domain of the porous medium using a numerical method. Typically, for a three-dimensional problem, one has to solve three independent flow problems with an averaged flow imposed by the boundary conditions in the way to ensure the independence of these three solutions. A first approach consists then in extracting the averaged velocity and pressure gradients. Then, one has to solve the linear system with nine equations and nine unknowns:

(21) $$\begin{array}{c} \begin{array}{c} \left[\begin{array}{c} K_{xx}\\ K_{xy}\\ K_{xz}\\ K_{yy}\\ K_{yz}\\ K_{zx}\\ K_{zy}\\ K_{zz} \end{array}\right]=\mu {\left[\begin{array}{ccccccccc} \nabla P_{x}^{1} & \nabla P_{y}^{1} & \nabla P_{z}^{1} & 0 & 0 & 0 & 0 & 0 & 0\\ 0 & 0 & 0 & \nabla p_{x}^{1} & \nabla p_{y}^{1} & \nabla p_{z}^{1} & 0 & 0 & 0\\ 0 & 0 & 0 & 0 & 0 & 0 & \nabla p_{x}^{1} & \nabla p_{y}^{1} & \nabla p_{z}^{1}\\ \nabla P_{x}^{2} & \nabla P_{y}^{2} & \nabla P_{z}^{2} & 0 & 0 & 0 & 0 & 0 & 0\\ 0 & 0 & 0 & \nabla p_{x}^{2} & \nabla p_{y}^{2} & \nabla p_{z}^{2} & 0 & 0 & 0\\ 0 & 0 & 0 & 0 & 0 & 0 & \nabla p_{x}^{2} & \nabla p_{y}^{2} & \nabla p_{z}^{2}\\ \nabla P_{x}^{3} & \nabla P_{y}^{3} & \nabla P_{z}^{3} & 0 & 0 & 0 & 0 & 0 & 0\\ 0 & 0 & 0 & \nabla p_{x}^{3} & \nabla p_{y}^{3} & \nabla p_{z}^{3} & 0 & 0 & 0\\ 0 & 0 & 0 & 0 & 0 & 0 & \nabla p_{x}^{3} & \nabla p_{y}^{3} & \nabla p_{z}^{3} \end{array}\right]}^{-1} \end{array}\left[\begin{array}{c} v_{x}^{1}\\ v_{y}^{1}\\ v_{z}^{1}\\ v_{x}^{2}\\ v_{y}^{2}\\ v_{z}^{2}\\ v_{x}^{3}\\ v_{y}^{3}\\ v_{z}^{3} \end{array}\right] \end{array}$$

where the subscripts *x*, *y*, and *z* denote directions, and the subscripts 1, 2, and 3 denote a numerical simulation run in each direction. The solution obtained by solving this matrix system is an approximate solution. For this reason, the symmetry of the permeability tensor identified at this stage is not always respected. In most cases, the calculated $K_{ij}$ components are not identical to the $K_{ji}$ components, where the ij indices denote x,y, and *z*. In such cases, to ensure the symmetry, it is common to make the following modification to the off-diagonal terms:

(22) $$\begin{array}{c} K_{ij}^{final}=K_{ji}^{final}=\frac{K_{ij}+K_{ji}}{2} \end{array}$$

The degree of asymmetry is also induced by the type of boundary conditions used to perform the calculation. The asymmetry reduces as the sample size increases because boundary effects contribute much less.

Another approach relies on homogenisation to connect the pore-scale flow and the continuum-scale flow [25]. Similar to homogenisation in micromechanics, where the Hill–Mandel lemma is used as an equivalence criterion between physical quantities at both scales, here, the total power dissipated by the flow at both scales is used. When the flow occurs through porous media, there is a dissipation of energy equal to the hydraulic pressure loss along the flow field. This energy loss is equal to the work of the viscous forces resisting the flow.

The powers at micro- and macro-scales are given by

(23) $$\begin{array}{c} P_{micro}=\int_{RV}\sigma :\nabla vdx \end{array}$$

(24) $$\begin{array}{c} P_{macro}=\nabla P\;\cdot \;V\left|w\left(X\right)\right| \end{array}$$

|w(X)| is called the measure of w(X). Following the derivation in [25], one obtains

(25) $$\begin{array}{cc} 2\mu \langle D:D\rangle & =\nabla P\;\cdot \;V \end{array}$$

(26) $$\begin{array}{cc} 2\mu \langle D:D\rangle & =V^{T}\;\cdot \;K^{-1}\;\cdot \;V \end{array}$$

where D is the rate of strain tensor. Thus, knowing the velocity fields (and subsequently the viscous dissipation) from the flow solutions, one can obtain the full permeability tensor from Equation (25). This method does not require the calculation of the pressure field in order to compute the permeability.

### 3.6. Boundary Conditions

Solving the equations of the heterogeneous pore flow model and those of the upscaled macroscopic flow model requires, in both cases, the definition of a set of boundary conditions (BC).

A commonly used condition is to reproduce those used in the laboratory to measure the permeability of a material. In the case of composite fiber reinforcements, the domain is a rectangular parallelepiped (rectangular cuboid). A constant pressure is applied to one face and a different pressure is applied to the opposite face, creating a constant pressure gradient; the other surfaces are impermeable (i.e., zero pressure gradient normal to their plane, no slip velocity). This boundary condition is sometimes called a permeameter boundary condition. An alternative is to impose a constant macroscale velocity on the two permeable boundaries. The main issue with this BC is that it suppresses important transverse flows and can result in unphysical flows. This problem is overcome by using periodic boundary conditions. These have been used widely in the framework of homogenisation theory. However, this condition rarely corresponds to the situation encountered with fibrous reinforcement stacks, where the bounding box of the Representative Volume Element (RVE) intersects the solid and fluid phases non-periodically.

Symmetry is an intermediate condition between the permeameter and periodic BCs. The velocities tangential to the bounding walls are allowed to evolve as if there were no walls, but all velocities normal to the walls are zero. This BC also suppresses transversal flow within the material but partially circumvents the no-flow boundary problem along all four faces of the computational domain in terms of diagonal permeability tensor terms by allowing non-zero velocities along the boundaries. The periodisation of non-periodic porous media can be achieved through translation or symmetry procedures [13]; the first one, however, creates a risk to provide a non-percolating geometry, while the second one increases the size of a computational domain by multiple times.

It has been observed that near the boundary of the computational domain, flow characteristics may better reflect prescribed physical flow constraints than the effects of local permeability. This can bias the expected directionality of fluid flow and therefore the predicted permeability. To mitigate this effect, it has been proposed to add a boundary region to impose boundary conditions further away from the boundaries by immersing the domain of interest in a larger domain with the same type of heterogeneity [13,26]. An equivalent approach is to average the calculated fields over a restricted sub-volume of the flow away from the boundaries. This approach of sub-volume immersion involves additional computational cost due to the presence of the border region.

### 3.7. Body Force-Driven Flow

A common element in minimizing the bias introduced by the non-representativeness of the processed digital samples and boundary conditions is to address the largest possible porous media in 3D, avoiding a periodisation strategy. The computational efficiency of the numerical technique and the ability to apply appropriate boundary conditions are central in this challenge. Similar to LBM, where a uniform body force can be used instead of a pressure gradient to produce the same flow rate as pressure-driven flow [27], body force-driven flow is used in PoroS. It has been shown that the permeability tensor asymmetry problem for very heterogeneous samples is reduced by forcing a flow with a body force into the sample and applying periodic boundary conditions [28].

In this work, for the calculation of permeability, a uniform unit body force is applied in an appropriate direction depending on the flow problem under consideration; i.e., for s flow problem in the X direction, $b_{x}=1,b_{y}=0,b_{z}=0$ is imposed; similarly, for flow problems in the Y and Z directions, $b_{x}=0,b_{y}=1,b_{z}=0$ and $b_{x}=0,b_{y}=0,b_{z}=1$ are imposed, respectively.

## 4. Validation of PoroS

This section discusses three test cases from the literature that serve as the validation of the PoroS solver for the Stokes flow solution. The first test case consists of a Poiseuille flow in a 3D pipe with Dirichlet-type boundary condition without considering the body force to validate the Stokes flow solver. The second and third test cases (Section 4.2 and Section 4.3) involve the calculation of the permeability using a body force approach. Their goal is to validate the entire approach on a simple geometry using a volumetric forcing condition to predict the permeability.

### 4.1. Poiseuille Flow in a 3D Pipe with Circular Cross Section

**Figure 2:**
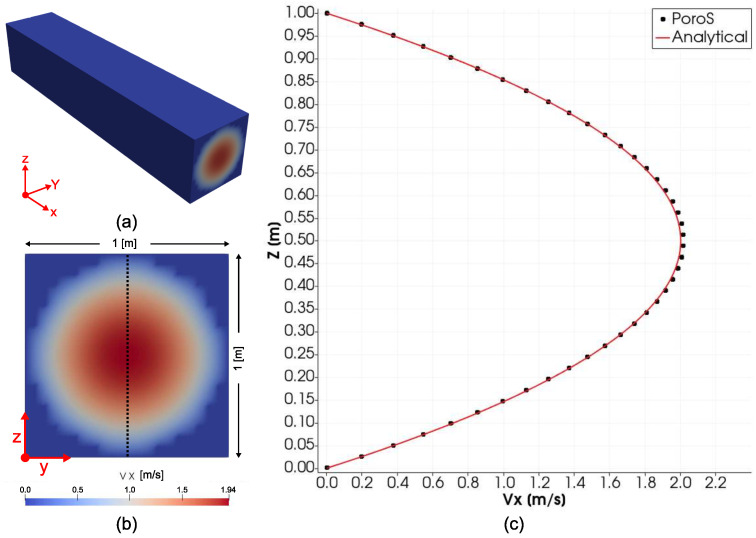
(**a**) Poiseuille flow in a 3D pipe with a circular cross section. (**b**) Cross sectional view of the pipe. (**c**) Comparison of the $V_{x}$ profile in the middle of the domain obtained using PoroS with the known analytical solution.

In order to validate the Stokes flow solver, a voxelized version of the classical test case of Poiseuille flow in a 3D pipe of a circular cross section was considered. Even though the cross sectional geometry was considered to be rectangular, v=0 was imposed on all nodes on the voxelized boundary of the cross section and all elements outside the boundary were set to solid regions, thereby restricting the flow outside the circular pipe (Figure 2a). The pipe has a length of 5 m and a diameter of 1 m. A total of 20 voxels were used across the diameter for discretisation. A uniform velocity (along X) of 1 m/s was applied at the circular inlet. After performing the simulation, the fully developed $V_{x}$ profile at the centre of the cross section (Figure 2b) was taken for comparison with the analytical solution discussed in [29]. A good match was observed between the numerical solution calculated using PoroS and the analytical solution (Figure 2c).

### 4.2. Validation of Prediction of Transverse Permeability of a Fiber Array

**Figure 3:**
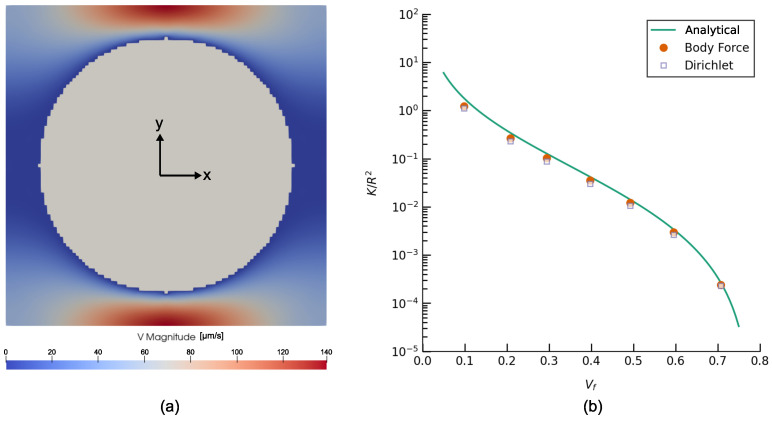
(**a**) Magnitude of velocity field in [μm/s] in case of a body force-driven flow ($V_{f}=0.493$). (**b**) Comparison of the normalized transverse permeability ($K_{xx}/R^{2}$) obtained using an analytical expression from [30], numerical simulations performed using a body force-driven flow, and numerical simulations performed using a Dirichlet condition-driven flow.

To validate the numerical prediction of permeability by PoroS of a microstructure in the form of array of aligned cylinders (fibres) transversely to their direction, a test from [30] has been considered. It involves a flow through an RVE of this microstructure with a quadratic packing, i.e., around a cylindrical obstacle depicted in Figure 3a. A voxelized geometry of 101×101×11 [μm] was considered with a resolution of 1 [μm/voxel]. A voxel is an equivalent of a pixel but in 3D space. It can be defined as a data point in a 3D regular grid. Several cases with different fibre volume fractions ($V_{f}$) were considered by varying the diameter of the cylindrical obstacle. Simulations were carried out considering (i) a Dirichlet condition-driven flow and (ii) a body force-driven flow without additional fluid buffer zone, both with a convergence criteria of 0.1% for the permeability. The $K_{xx}$ component of the permeability tensor normalized with the square of the radius (*R*) of the cylinder for all the cases is plotted in Figure 3b that shows a good match with the analytical solution discussed in [30].

### 4.3. Validation of Prediction of Longitudinal Permeability

**Figure 4:**
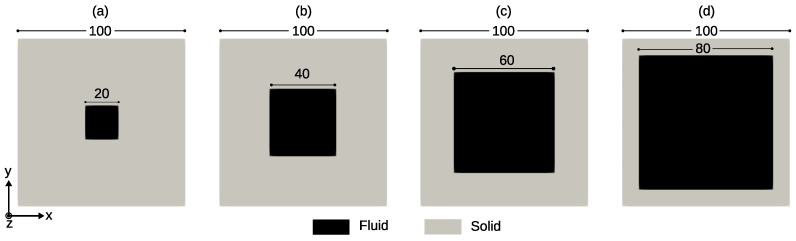
Geometry of the models for the calculation of the longitudinal permeability: a square channel of cross section: (**a**) 20×20 [μm]; (**b**) 40×40 [μm]; (**c**) 60×60 [μm]; (**d**) 80×80 [μm].

To validate the numerical prediction by PoroS of the longitudinal permeability, a test inspired by [31] has been considered. It involves a flow in the domain containing a channel with a constant square cross section, and the volume fraction of this channel within the domain is being varied (Figure 4). A geometry of dimensions 100×100×100 [μm] was considered with a resolution of 1 [μm/voxel]. Four sub-cases of this test were considered where different diameters of the inner square channel were considered: 20, 40, 60, and 80 [μm] as shown in Figure 4.

The problem was solved for a fluid of viscosity 1 [Pa·s], and a penalty number of 1000 was used for imposing the incompressibility constraint. The simulations were carried out by considering the body force-driven loading. The numerical results obtained using PoroS were compared to the empirical results presented in [32], where the equivalent longitudinal permeability of the domain with channels with a square cross section is given by the following:

(27) $$\begin{array}{c} K_{\mathrm{empirical}}=0.035144\frac{l^{4}}{A} \end{array}$$

where *l* is the channel cross section edge length and *A* is the full cross section area, including the solid region.

It is evident from the comparison (Table 1), that the numerical prediction of the permeability is in very good agreement with the corresponding empirical values.

The validation using these three conventional test cases served for testing not only the solver but also the new finite element-based approach using a volumetric forcing condition to predict the permeability. This first step of validation is essential before proceeding to compare the permeability prediction results on a much bigger RVE from the international virtual permeability benchmark. This is discussed in the next section.

## 5. Results and Discussion

This section consists of two parts. In Section 5.1, the results computed by PoroS are compared to the results from the international virtual permeability benchmark on fibrous microstructures. In Section 5.2, the importance of a correct computation of a full permeability tensor including the off-diagonal terms is discussed illustrating it, first, with a simple example of a 2D inclined channel network, and, second, with a real 3D microstructure.

### 5.1. Comparison of Permeability Values Obtained by PoroS with the Results from the International Virtual Permeability Benchmark

**Figure 5:**
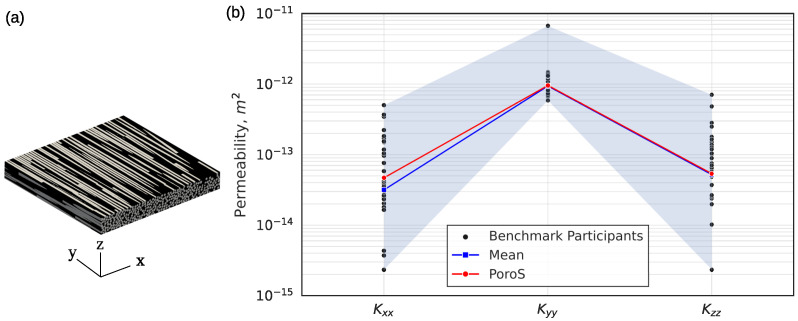
(**a**) The 3D segmented image used as an input for the international virtual permeability benchmark. (**b**) Comparison of results obtained with PoroS solver with body forcing with respect to all the results of the benchmark participants.

The first international virtual permeability benchmark conducted for engineered textiles [3] has discussed and compared various numerical modelling approaches in detail. The benchmark has provided 50 values computed by 16 participants for the same input geometry (a 3D segmented image of the representative volume element), which can be found in the repository [33]. It was obtained from the 3D X-ray microscope scan of a twill-weave glass fibre reinforced composite (HexForcce 01102 from HEXCEL, Dagneux, France) (Figure 5a). The input was a 3D voxelised image with dimensions of 1003 × 124 × 973 voxels and a scan resolution of 0.521 [μm/voxel] in each direction. This problem with a total of about 120 million voxels resulted in a total of about 3 billion DoFs.

In the following sections, three comparative studies are undertaken where the results are compared for the following: the full geometry, the geometry divided into 10 sub-volumes across the fibre direction, and finally, the geometry divided into 10 sub-volumes along the fibre direction.

#### 5.1.1. Comparison with the Results on the Full Geometry of the RVE

The results obtained with PoroS 1.0 using the body forcing fall within the cluster of the results of the participants of the benchmark (Figure 5b). Additionally, the results are very close to the mean values of the benchmark results, which were obtained after eliminating the outliers (refer to [3] for more details on how the means were obtained).

This indicates that the approach of using body force-driven flow in a microstructure provides good results for the permeability of a heterogeneous microstructure, which is consistent with the results obtained using different modelling techniques reported by various participants in the benchmark.

#### 5.1.2. Comparison with the Results on the Sub-Volumes: Transverse Cuts

**Figure 6:**
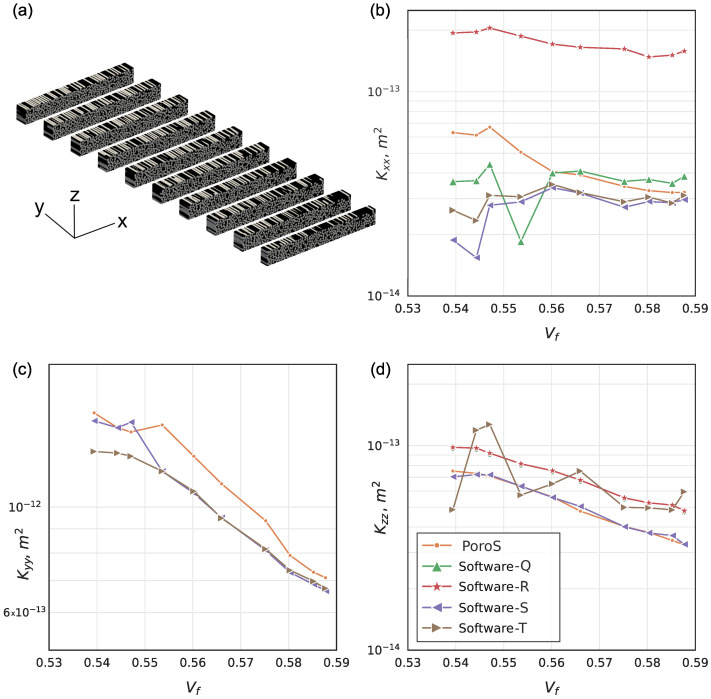
Comparison with the benchmark results: transverse sections (**a**); $K_{xx}$ (**b**); $K_{yy}$ (**c**); $K_{zz}$ (**d**).

Further analysis was conducted to ensure the validity of this approach by comparing the permeability results computed on 10 sub-volumes of the RVE of the benchmark, where the RVE was subdivided by slicing the geometry transverse to the fibre direction, as shown in Figure 6a. With such a subdivision, a monotonic evolution of the fibre volume fraction along the fibre direction can be observed within this sample: it increases from 0.54 to 0.59 along the fibre direction. This evolution is due to the woven geometry of the fabric where warp and weft tows intersect and thus can be locally more compacted at these crossings. The natural decrease in permeability with increasing $V_{f}$ in this case was not always correctly captured by the benchmark participants, who conducted this study on sub-volumes [3]. For the longitudinal component of the permeability $K_{yy}$ in the direction of the fibres, i.e., when the flow has straighter and less complex paths through the geometry, this trend was correctly captured by all calculations; PoroS also showed a correct trend (Figure 6c). However, predicting consistent values of $K_{xx}$ with respect to $V_{f}$ was challenging for the benchmark participants (Figure 6b), while PoroS succeeded in predicting the decreasing permeability trend. The only exception to the decreasing trend was a higher $K_{xx}$ value calculated on sub-volume 3 with $V_{f}=0.547$, which was also detected by Software-Q and Software-R (Figure 6b). This value can be explained by the fact that with a $V_{f}$ close to the previously calculated $V_{f}=0.544$, this sub-volume had a locally very open channel with the dominant effect on permeability.

It was noted in [3] that a correct dependence of the Software-R result on the fibre volume fraction was obtained thanks to the permeability identification procedure, which allows the computation of the full permeability tensor, which is also the case for PoroS (this point will be discussed in more detail in Section 5.2). Quantitatively, however, the $K_{xx}$ result of Software-R is too far away from the cluster of consistent benchmark results, while the values predicted by PoroS correlate well with the cluster. The results of Software-T also yielded the full tensor but were classified as non-consistent in the benchmark due to the numerical methodology used.

For the transverse permeability component $K_{zz}$, more participants were able to predict a correct decreasing trend (Figure 6d), because numerically, the flow had much simpler paths through the small thickness of the domain in this direction compared to the case of $K_{xx}$. PoroS also produced predictions very close to other participants’ values.

#### 5.1.3. Comparison with the Results on the Sub-Volumes: Longitudinal Cuts

**Figure 7:**
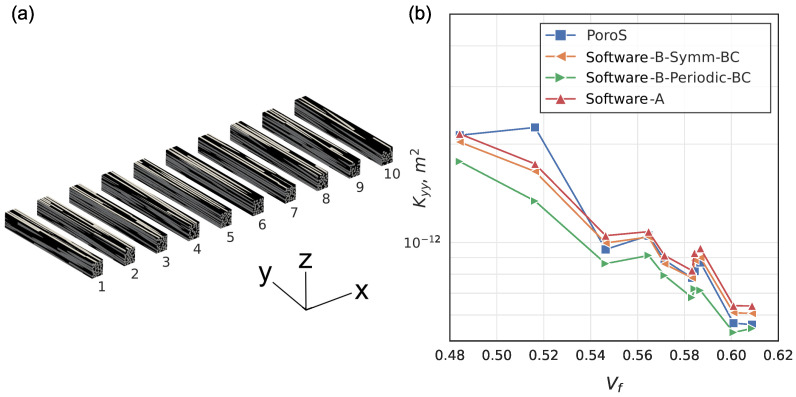
Comparison with the benchmark results: (**a**) longitudinal sections; (**b**) $K_{yy}$.

The main motivation for the participants to conduct the study on the sub-volumes of the RVE of the benchmark was the significant computational cost associated with handling the whole RVE. From the computations on the transverse sections considered in the previous section, the participants derived the equivalent transverse permeability components of the entire RVE. Similarly, they used longitudinal slices of the RVE (parallel to the fibre direction; Figure 7a) to calculate the equivalent axial permeability component $K_{yy}$ considered in this section.

On the contrary, as shown previously, PoroS was not limited by memory capacity in predicting the permeability of the entire RVE. Its study on the cross sectional volumes was performed to prove its ability to correctly capture the permeability dependence on the fibre volume fraction compared to the approaches used in the benchmark. Contrary to the direction along the fibres with their gradual compaction and thus monotonic change in $V_{f}$, the longitudinal sub-volumes did not have such trend in $V_{f}$ change, since they were distributed along the width of the tow. However, locally, the fibre volume fraction still varied from sub-volume to sub-volume (Figure 7a) due to the misalignment and twisting of the fibres within the tow. The results of the predicted permeability $K_{yy}$ on 10 sub-volumes are plotted against increasing $V_{f}$ in Figure 7b. The general trend of decreasing permeability is mainly observed in the results of the indicated software and in the results of PoroS. It should be noted that, unlike the transverse slices of the previous section, the longitudinal slices each contain a very small number of fibers that are not statistically representative of the entire microstructure. This can explain the fluctuations in the decreasing trend of the permeability, especially for close values of $V_{f}$. Moreover, all results in Figure 7b follow the same trend and are close to each other. The exception is the $K_{yy}$ value predicted by PoroS at $V_{f}=0.516$. This small $V_{f}$ corresponds to the boundary sub-volume 10 (refer to Figure 7a), which has a peculiar geometry, with some fibres strongly misaligned with respect to the axial Y direction. The determination of the full permeability tensor via homogenisation in PoroS allowed us to capture these effects. This point is further discussed in Section 5.2.2.

The benchmark [3] demonstrated the importance of boundary conditions applied tangentially to the flow direction. For this tow microstructure, it was shown that the application of the symmetric BC resulted in higher axial permeability $K_{yy}$ values than the periodic BC. The same is confirmed for the sub-volume study: the results of Software-A also used symmetric BC in Figure 7b. This tendency can be explained by the geometry of this microstructure due to some channels at the boundaries, which are external to the whole RVE and which double their size in the case of symmetry application, thus opening large flow paths in the axial direction. These channels do not increase in size when periodicity is applied. Since PoroS is a body force-driven flow, as opposed to conventional boundary conditions, the majority of the values in Figure 7b computed by PoroS lie between the values of periodic BC and symmetric BC. This fact underlines another advantage of the method implemented in PoroS: in domains with a strong influence of boundary conditions, the body force approach solves the problem of their adequate choice.

In conclusion, the observations discussed in these comparative sections indicate that the approach of using body force-driven flow through a microstructure results in a good prediction of its permeability, and this was demonstrated starting with academic test cases from the literature and then further through comparisons with the results from the virtual permeability benchmark. This completes the first objective of this article. The second objective, which is to demonstrate the need to calculate the full permeability tensor including its off-diagonal components, is discussed next.

### 5.2. Importance of Predicting the Full Permeability Tensor

To demonstrate the importance of predicting the full permeability tensor of the structure, two test cases are presented. Section 5.2.1 discusses a representative test case that has been specially constructed. Section 5.2.2 revisits the longitudinal sub-channel 10 to discuss the peculiarities of the flow field computed within it.

#### 5.2.1. A Case with a 2D Inclined Channel Network

**Figure 8:**
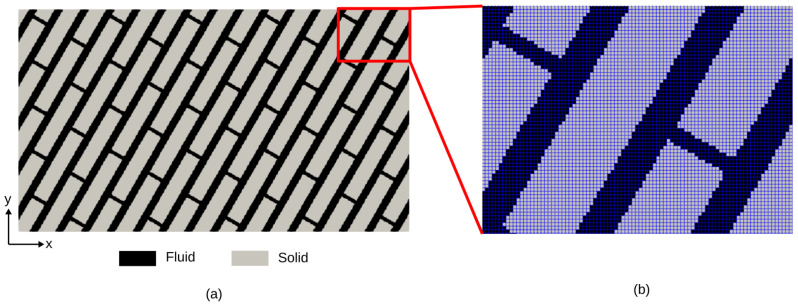
(**a**) Geometry of a 2D channel network inclined at $60^{\circ }$. (**b**) Voxelized zoomed view of geometry.

This representative test case consists of a 2D orthogonal channel network where wider channels are inclined at an angle of $60^{\circ }$ with respect to the X axis (Figure 8a). PoroS uses a pixelized image in 2D (500×285 pixels in X and Y directions for this test case with a resolution of 1 [μm/px]) as an input, where one pixel corresponds to one element, as shown in Figure 8b.

**Figure 9:**
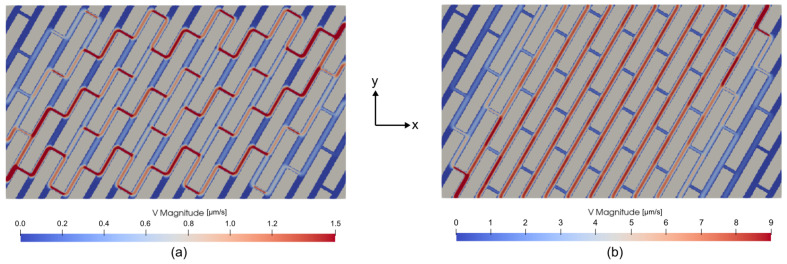
Field of |V| in [μm/s] for (**a**) flow problem in X and (**b**) flow problem in Y.

The steady-state Stokes flow problems in the X and Y directions were solved using PoroS solver with the body force approach, as described in Section 3.1. The field of magnitude of velocity for X and Y flow problems is shown in Figure 9.

After performing the homogenization of the structure, the macroscopic permeability tensor was calculated to be

(28) $$\begin{array}{c} \left[\begin{array}{cc} K_{11} & K_{12}\\ K_{21} & K_{22} \end{array}\right]=\left[\begin{array}{cc} 4.41\times 10^{-13} & 6.66\times 10^{-13}\\ 6.66\times 10^{-13} & 1.20\times 10^{-12} \end{array}\right] \end{array}$$

It is evident from the relative magnitudes of the off-diagonal terms that they cannot be neglected for such a structure. Going further, the principal flow directions for this case can be obtained by calculating eigenvalues ($\lambda_{i}$) and eigenvectors ($V_{i}$) of the permeability tensor in Equation (28), which are

(29) $$\begin{array}{c} \lambda_{1}=5.45\times 10^{-14},\;V_{1}=\left[\begin{array}{c} -0.87\\ 0.50 \end{array}\right]=149.91{}^{\circ};\;\lambda_{2}=1.59\times 10^{-12},\;V_{2}=\left[\begin{array}{c} 0.50\\ 0.87 \end{array}\right]=59.91{}^{\circ} \end{array}$$

Angles calculated in Equation (29) provide the validation that the main flow direction (corresponding to the highest eigenvalue, i.e., $\lambda_{2}$) is along 59.91°, which matches with the orientation of the wider channels (60°).

#### 5.2.2. The Importance of Identifying the Dominant Flow Direction

The calculation of the skew terms of the permeability tensor becomes essential for the materials with the architecture for which the flow principal directions are unknown. For instance, the 3D woven fabric studied in [34] exhibited an asymmetric flow in the thickness direction due to its complex weaving pattern. This effect was clearly reflected on the non-negligible order of magnitude of the measured off-diagonal components of its permeability tensor.

**Figure 10:**
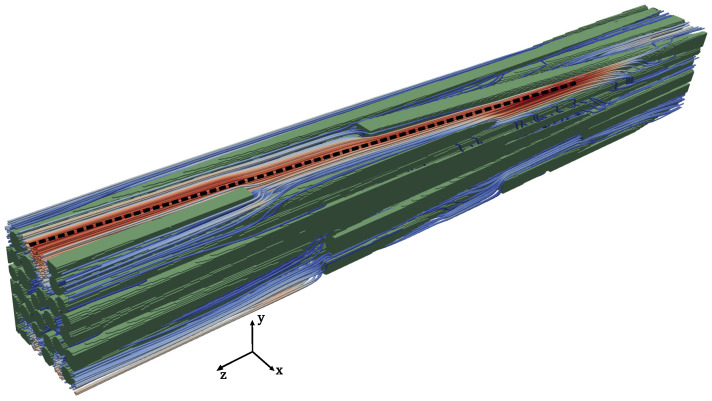
Local fibres’ misalignment changing the flow principal directions in the case of the longitudinally cut sub-volume 10 (refer to Figure 7a) from the international virtual permeability benchmark case.

For a typical quasi-isotropic or orthotropic material, it might be sufficient to just evaluate the diagonal components of the permeability. However, the permeability of the anisotropic materials will be determined by the principal flow paths and their directions. This principal flow path will often be driven by the orientations of the material itself (as demonstrated in Section 5.2.1), but it also depends on the dominant flow direction which may or may not be the same as that of the material orientation. For example, an interesting flow pattern was observed while considering the longitudinally cut sub-volume 10 (refer to Figure 7a) from Section 5.1.3 while looking at the flow problem Z in the axial direction of fibres. Here, even though the majority of the fibres are indeed oriented along the Z direction, the dominant flow direction is different from the Z direction (indicated by the dotted line in Figure 10). This is due to a strong misalignment of some fibres.

It was observed that the ratio $K_{zz}/K_{xz}$ was high for most of the longitudinally cut sub-volumes of the benchmark (going as high as 1600 for sub-volume 6). Meanwhile, in the case of sub-volume 10, it was found to be up to 44. Thus, even though one can know the overall material direction of a microstructure under consideration, it is not obvious that the dominant flow direction would be along this direction of the material. This emphasises the need for evaluating the full permeability tensor, including its off-diagonal components. These effects are visible on a large scale during the manufacture of composite parts but not necessarily on too small digital samples. It is therefore important to ensure the representativeness of the latter in order to predict these directional effects and the resulting cross-flows.

## 6. Conclusions

Measuring the permeability of heterogeneous porous media is difficult and can lead to significant errors. While many approaches have been established to predict the permeability of homogeneous porous media, the permeability of anisotropic and heterogeneous porous media such as fibrous media and cracked rocks remains a real challenge. The numerical determination of permeability is therefore an important issue in many engineering sectors. The difficulty of the problem lies primarily in the ability to handle numerical samples of sufficient size to account for the statistical properties of the media that become apparent at certain scales. Second, we need to reduce the effects of boundary conditions, which can lead to unphysical flows.In response to these challenges, a novel finite element-based approach for predicting the permeability of heterogeneous and anisotropic porous microstructures was proposed where the flow in the microstructure is induced by a body force. The novelty lies in the combination of the volumetric forcing condition, an optimized finite element solver, and the calculation of the complete permeability tensor by homogenisation without pressure calculation. In order to investigate the applicability, accuracy, and numerical efficiency of this approach, a new solver suite called PoroS 1.0 was developed that consists of Stokes equation flow solvers and the permeability calculation program. It has image-based FEM solvers that work directly on the segmented images of the microstructure obtained from either the micro-CT scan of the material or using the digital twin of the material generated using the widely used TexGen^®^ software. The architecture of PoroS has been specifically designed to work with a large number of degrees of freedom (order of billions), which is often the case for such voxelized models generated from the scans.

To prove that the body force-driven flow condition is valid, PoroS was first validated with various standard flow and permeability problems from the literature, such as Poiseuille flow in a pipe and 2D flow in channels with square and rectangular obstacles. Then, a detailed comparison was made with respect to the results of the international numerical permeability benchmark on fibrous media, thereby validating both the solver and the novel approach. This challenging test case has a total of about 120 million voxels, resulting in a total of about 3 billion DoFs. By comparing results from other software, it is shown that in domains with a strong influence of boundary conditions, the body force-driven flow leads to more accurate predictions. The method of computing the complete tensor by homogenisation, combined with the ability to process the complete geometry, leads to more accurate results than approaches that require the geometry to be subdivided or that do not identify the full tensor. Finally, the importance of predicting the full permeability tensor was also demonstrated, especially in the case of heterogeneous and anisotropic porous microstructures, where the dominant flow direction might be different from the dominant material structure orientation.

As a perspective of the future, a version 2.0 of PoroS has been under development that will address the dual-scale flow problems using the resolution of Stokes–Brinkman equations while also taking into account the local orientations of the porous microstructure. It will retain all advantages of PoroS 1.0, while introducing new features in order to handle more advanced multi-scale problems in numerical permeability estimation.

## Figures and Tables

**Table 1:** Comparison of the numerical predictions of the longitudinal permeability with the empirical results from the literature [32].

Case	$K_{\mathrm{empirical}}$	$K_{\mathrm{numerical}}$	%Error
1	5.62304 × 10^−1^	5.62310 × 10^−1^	0.0011%
2	8.99686	8.99690	0.0004%
3	4.55466 × 10^1^	4.55470 × 10^1^	0.0008%
4	1.43950 × 10^2^	1.43950 × 10^2^	0.0001%

## Data Availability

The raw data supporting the conclusions of this article will be made available by the authors on request.

## Abbreviations

The following abbreviations are used in this manuscript:

RVE	Representative Volume Element
BC	Boundary Conditions
DoF	Degree of Freedom
FEM	Finite Element Method
FVM	Finite Volume Method
FDM	Finite Difference Method
PNM	Pore Network Model
SPH	Smooth Particle Hydrodynamics
LBM	Lattice Boltzman Method

## References

[1] Shojaei A., Trochu F., Ghaffarian S., Karimian S., Lessard L. (2004). An experimental study of saturated and unsaturated permeabilities in resin transfer molding based on unidirectional flow measurements. J. Reinf. Plast. Compos..

[2] Papán D., Deckỳ M., Ďugel D., Durčák F. (2024). Identification of Hybrid Polymer Material STERED and Basic Material Properties Used in Road Substructures or Pavements. Polymers.

[3] Syerko E., Schmidt T., May D., Binetruy C., Advani S., Lomov S., Silva L., Abaimov S., Aissa N., Akhatov I. (2023). Benchmark exercise on image-based permeability determination of engineering textiles: Microscale predictions. Compos. Part A Appl. Sci. Manuf..

[4] Gambarini G., Valdés-Alonzo G., Binetruy C., Comas-Cardona S., Syerko E., Waris M. (2024). Directional saturation of a strongly bimodal pore size distribution carbon interlock fabric: Measurement and multiphase flow modeling. Compos. Part B Eng..

[5] Sun Z., Logé R.E., Bernacki M. (2010). 3D finite element model of semi-solid permeability in an equiaxed granular structure. Comput. Mater. Sci..

[6] Osorno M., Uribe D., Ruiz O.E., Steeb H. (2015). Finite difference calculations of permeability in large domains in a wide porosity range. Arch. Appl. Mech..

[7] Blunt M.J. (2001). Flow in porous media—pore-network models and multiphase flow. Curr. Opin. Colloid Interface Sci..

[8] Eshghinejadfard A., Daróczy L., Janiga G., Thévenin D. (2016). Calculation of the permeability in porous media using the lattice Boltzmann method. Int. J. Heat Fluid Flow.

[9] Belov E., Lomov S., Verpoest I., Peters T., Roose D., Parnas R., Hoes K., Sol H. (2004). Modelling of permeability of textile reinforcements: Lattice Boltzmann method. Compos. Sci. Technol..

[10] Pereira G.G., Dupuy P.M., Cleary P.W., Delaney G.W. (2012). Comparison of permeability of model porous media between SPH and LB. Prog. Comput. Fluid Dyn. Int. J..

[11] Wagner A., Eggenweiler E., Weinhardt F., Trivedi Z., Krach D., Lohrmann C., Jain K., Karadimitriou N., Bringedal C., Voland P. (2021). Permeability estimation of regular porous structures: A benchmark for comparison of methods. Transp. Porous Media.

[12] Bancora S., Binetruy C., Advani S., Comas-Cardona S., Leygue A. (2023). Efficient dual-scale flow simulation for Resin Transfer Molding process based on domains skeletonization. Compos. Part A Appl. Sci. Manuf..

[13] Guibert R., Horgue P., Debenest G., Quintard M. (2016). A Comparison of Various Methods for the Numerical Evaluation of Porous Media Permeability Tensors from Pore-Scale Geometry. Math. Geosci..

[14] Donea J., Huerta A. (2003). Finite Element Methods for Flow Problems.

[15] Hughes T.J., Liu W.K., Brooks A. (1979). Finite element analysis of incompressible viscous flows by the penalty function formulation. J. Comput. Phys..

[16] Heinrich J., Marshall R. (1981). Viscous incompressible flow by a penalty function finite element method. Comput. Fluids.

[17] Temam R. (2001). Navier-Stokes Equations: Theory and Numerical Analysis.

[18] David Müzel S., Bonhin E.P., Guimarães N.M., Guidi E.S. (2020). Application of the finite element method in the analysis of composite materials: A review. Polymers.

[19] Lubecki M., Stosiak M., Karpenko M., Urbanowicz K., Deptuła A., Cieślicki R. (2023). Design and FEM analysis of plastic parts of a tie-rod composite hydraulic cylinder. Mechanics.

[20] Long A., Brown L. (2011). Modelling the geometry of textile reinforcements for composites: TexGen. Composite Reinforcements for Optimum Performance.

[21] Brown L., Matveev M., Spackman G. (2023). Louisepb/TexGen: TexGen v3.13.1.

[22] Taylor C., Hood P. (1973). A numerical solution of the Navier-Stokes equations using the finite element technique. Comput. Fluids.

[23] Sánchez-Palencia E. (1980). Non-homogeneous media and vibration theory. Lect. Note Phys..

[24] Whitaker S. (1986). Flow in porous media I: A theoretical derivation of Darcy’s law. Transp. Porous Media.

[25] Lopez E., Abisset-Chavanne E., Lebel F., Upadhyay R., Comas S., Binetruy C., Chinesta F. (2016). Flow modeling of linear and nonlinear fluids in two and three scale fibrous fabrics. Int. J. Mater. Form..

[26] Wen X., Durlofsky L., Edwards M. (2003). Use of border regions for improved permeability upscaling. Math. Geol..

[27] Nabovati A., Llewellin E.W., Sousa A.C. (2009). A general model for the permeability of fibrous porous media based on fluid flow simulations using the lattice Boltzmann method. Compos. Part A Appl. Sci. Manuf..

[28] Zakirov T., Khramchenkov M. (2022). Study of the pore space heterogeneity effect on the absolute permeability tensors calculated under different boundary conditions and driving forces using a “Computational Rock Physics” technology. J. Pet. Sci. Eng..

[29] White F.M. (1979). Fluid Mechanics.

[30] Gebart B.R. (1992). Permeability of unidirectional reinforcements for RTM. J. Compos. Mater..

[31] Saxena N., Hofmann R., Alpak F.O., Berg S., Dietderich J., Agarwal U., Tandon K., Hunter S., Freeman J., Wilson O.B. (2017). References and benchmarks for pore-scale flow simulated using micro-CT images of porous media and digital rocks. Adv. Water Resour..

[32] Mavko G., Mukerji T., Dvorkin J. (2020). The Rock Physics Handbook.

[33] Syerko E. (2022). International Virtual Permeability Benchmark 3D Image Dataset of the Fiber Tow Microscopic Sample.

[34] Yun M., Sas H., Simacek P., Advani S.G. (2017). Characterization of 3D fabric permeability with skew terms. Compos. Part A Appl. Sci. Manuf..

